# Autism Spectrum Disorder Traits and Other Offending Risk Factors in a London-Based Forensic Youth Population

**DOI:** 10.3390/children12101300

**Published:** 2025-09-25

**Authors:** Maria Loizidou, Alexandra Busse, Rebecca Lane, Sophie Marshall

**Affiliations:** 1 Forensic Child and Adolescent Mental Health Service (FCAMHS) at the Portman Clinic (North, Central and East London), The Tavistock and Portman NHS Foundation Trust, London NW3 5NA, UK; 2 Department of Clinical Psychology and Psychological Therapies, Norwich Medical School, University of East Anglia, Norwich NR4 7TJ, UK; 3 Cambridgeshire and Peterborough NHS Foundation Trust, Cambridge CB21 5EF, UK

**Keywords:** adverse childhood experiences, autism spectrum disorder, youth offending, mental health, neurodiversity, trauma, violence

## Abstract

Background: Research exploring the context in which ASD and offending are associated is limited, despite stereotyped perceptions that individuals with ASD are more violent than their typically developing peers. To address this gap, this research explored the influence of ASD, mental health and behavioural presentation, adverse childhood experiences, and demographic characteristics on offending presentations in a forensic youth sample. Methods: This was a cross-sectional study of a retrospective cohort, utilising secondary data of 327 young people from a forensic London-based service (83% male, *M*_age_ = 14.9 years old, SD = 1.90). Results: One hundred forty-two of these young people presented with either confirmed or suspected ASD diagnoses (83.8% male, *M*_age_ = 14.8 years old, SD = 1.84). Five offending categories (violence, sexually harmful behaviour, drug possession/supply, gang involvement, theft) and 32 offending behaviours were analysed in total. Poisson and negative binomial regression analyses indicated that ASD traits only increased the rate ratio of violent offences (*p* = 0.036) and gang involvement (*p* = 0.002). The use of substances significantly increased the rate ratio of theft (*p* = 0.012), gang involvement (*p* = 0.004), and drug possession/supply (*p* = 0.012). Conclusions: Our findings suggest that ASD, in the context of substance use, may increase a young person’s risk for more variable violent offences or gang involvement. Findings are discussed in the context of current research and recommendations for clinical practice and future research are made.

## 1. Introduction

Research has overwhelmingly disproven the misconceptions that individuals with autism spectrum disorder (ASD) are more violent or aggressive than others. Additionally, when individuals with ASD do offend, they do not necessarily engage in extreme violence at the rate of neurotypical offenders [1]. However, a consideration of the circumstances under which people with ASD may be at increased risk of offending, whether biological (e.g., psychiatric comorbidities) or environmental (e.g., adverse childhood experiences (ACEs)), is critical to their reintegration in the community.

When people with ASD engage in offending behaviour, this is often related to the experience of violent obsessions or fantasies; behavioural rigidity which might escalate to aggression; the pursuit of obsessional interests without realising their social implications; and a heightened vulnerability to coercion, exploitation, or radicalisation [2,3]. Some authors suggest that offenders with ASD are more likely to commit interpersonal crimes, as opposed to drug-related offences or crimes against property [4]. Based on this evidence, ASD might be considered a risk factor for certain types of crimes, although the influence of other predictors is only starting to be explored [5].

### 1.1. Comorbid Conditions

Children with intellectual disabilities and/or ASD are more likely to have been abused and therefore more likely to perpetrate abuse [6,7]. Further, some evidence suggests that variation in offending typology may be related to the perpetrator’s cognitive profile, with interpersonal crimes more likely to be committed by people on the higher end of the spectrum and/or those with more comorbid psychiatric conditions [4,8]. In fact, incidence of comorbid psychiatric conditions is higher among those with high functioning ASD [9]. However, people on the highest end of the spectrum are also more likely to experience situational risk factors that peers with lower cognitive abilities would not find themselves in, such as living in the community independently [10].

### 1.2. Adverse Childhood Experiences (ACEs)

Children with ASD have been found to experience more ACEs and abuse than their typically developing peers, both within the general population and in forensic settings [7,11]. The term ACEs was first introduced by Felitti and colleagues [12] to describe seven types of adversity (psychological/physical/sexual abuse, violence against mother, household mental health, household substance misuse, household incarceration), showing how their experience during childhood was related to poor health outcomes in adult life. Studies have linked ACEs with internalising (e.g., anxiety) and externalising (e.g., aggression) behavioural problems in primary school children [13].

### 1.3. Social Factors

The impact of lower socioeconomic status and social deprivation further increases the likelihood of experiencing more ACEs and, in turn, the risk of offending [14]. The impact of social deprivation may be particularly prominent in London, which presents with significant deprivation and a diverse ethnic population [15,16]. Therefore, it remains unclear whether the impact of ACEs and social deprivation will further increase the risk of offending in young people (YP) who present with a history of ACEs and ASD.

### 1.4. Objectives

Research has primarily focused on adults with ASD in countries with considerably improved social infrastructure and a more homogenous ethnic profile, which may not be relevant to London’s diverse and deprived population [6,10]. It is, however, critical to consider the cumulative influence of factors, including neurodiversity, ACEs, mental health, and demographic characteristics, which have not yet been considered in childhood populations [5]. This exploratory research aims to identify how sociodemographic variables and mental health presentation may contribute to ASD becoming a risk factor for more variable offending in a London-based forensic community sample. Addressing this literature gap is critical in designing effective and timely interventions to prevent youth offending.

## 2. Materials and Methods

### 2.1. Participants

Secondary data of YP that referred to community Forensic Child and Adolescent Mental Health Service (FCAMHS) at the Tavistock and Portman NHS Trust in London and which were gathered between the inception of the service (2018) and 2023 were considered. The FCAMHS service model is described in detail elsewhere [17]. YP who are referred to this team typically reside in the community and have had contact with youth justice services, mental health services and/or social services. To be eligible for support from the FCAMHS team, YP must be receiving support from professionals in one of the following London boroughs: Barnet, Camden, Islington, City, Hackney, Tower Hamlets, Haringey, Enfield, Waltham Forest, Newham, Redbridge, Barking and Dagenham, and Havering. Some of these boroughs have been found to present with significant resident deprivation according to the CENSUS 2021, as measured by household employment, level of education, health and disability, and housing conditions [16].

Consent was obtained from referrers at the point of referral. Consent specific to this research was not required, as it makes use of routine data originally collected for outcome monitoring purposes which are anonymised at the point of collection. This research is compliant with both national and trust-wide data privacy procedures.

### 2.2. Inclusion Criteria

The following inclusion criteria were considered: (1) YP under or recently turned 18 years old (at time of referral), (2) referred to FCAMHS at Tavistock and Portman NHS Trust, and who (3) presented with risk of engaging or forensic behaviours. Rejected referrals were included if they fulfilled the above eligibility criteria, even if beyond the catchment area of the team, or when the reason for rejection was related to requesting an intervention that the service was not commissioned to provide (e.g., individual therapy). It was not necessary for the included YP to present with any mental health concerns. Since 2018 the FCAMHS team received 353 referrals, with an acceptance rate of 91% (322 referrals).

The research inclusion criteria encompassed 327 YP (83% male, *M*_age_ = 14.9 years old, SD = 1.90), 142 of whom presented with ASD traits (83.8% male, *M*_age_ = 14.8 years old, SD = 1.84), and 185 of whom did not (82.7% male, *M*_age_ = 14.9 years old, SD = 1.95).

### 2.3. Materials

This research utilised patient data that were obtained for outcome monitoring purposes through electronic records and professional discussions. All information was de-identified during data collection. A detailed list of the information collected for outcome monitoring purposes, including this research, is presented in Appendix A.

### 2.4. Procedure

Upon referral, an anonymised identifier was created for each referred person, with demographic information (age, gender, ethnicity), neurodevelopmental profile, mental health and behavioural presentation, ACEs, and offending behaviours entered on a Microsoft Excel spreadsheet (Version 2208). Ethnic background was coded based on the ethnicity codes used across the National Health Services in the UK [18]. YP were assigned a numerical identifier at the point of entry on the spreadsheet; YP name, borough of residence, or other identifying information were not included. The spreadsheet was stored on a secure, trust-shared drive, which was password protected and with access granted only to clinicians involved in outcome monitoring. The spreadsheet was continually updated with new referrals and/or as new information became available, from February 2023 until May 2023, when the statistical analysis began.

### 2.5. Neurodevelopmental Profile

The following neurodevelopmental conditions were rated as ‘confirmed,’ ‘suspected’ (e.g., some evidence for the condition/in the process of diagnostic assessment), or ‘absent,’ according to available reports: ASD, attention deficit hyperactivity disorder (ADHD), learning disability, learning difficulty. To improve ecological validity, and due to the lengthy waiting lists for ASD assessments and the need for substantial autistic traits and indicative developmental history markers for a person to be referred, YP with suspected and confirmed ASD diagnoses were analysed as a single category [19]. The same principle was applied for ADHD, learning disability, and learning difficulty. YP with a confirmed (*n* = 75) or suspected (*n* = 68) diagnosis of ASD are hereafter referred to as YP with ‘ASD traits.’ Due to the nature of the community Forensic Child and Adolescent Mental Health Service (FCAMHS), neurodevelopmental diagnostic assessments were not always available to review.

### 2.6. Adverse Childhood Experiences Score

ACE score was based on the ten ACE categories (physical/verbal/sexual abuse, physical/emotional neglect, parental separation, domestic abuse, household mental health, household substance misuse, household incarceration) [20]. Information on YP’s histories was gathered through electronic records (e.g., social care chronologies) or clinical consultations. Referrers did not directly report on ACE scores, although YP’s adverse experiences became evident during consultations. One point was given following evidence of a single or prolonged traumatic event/experience that would fit the above categories, for instance mention of a neglectful home environment during childhood. Due to the nature of the service, it was not possible to obtain this information directly from the YP. To ensure reliability in this process ACE score was reviewed independently by authors ML, AB, SM, as well as the treating clinician within the team, who may have been more familiar with the case.

### 2.7. Offending Score

Offending score was calculated based on routine data available. The following offending categories were rated: violence, sexually harmful behaviour (SHB), gang involvement, drug possession/supply, radicalisation, arson, theft, stalking/harassment, other offences (free-text option). No other offences were specified in the ‘other offences’ category, which was subsequently excluded from analyses. The above offending categories included more specific presentations, totalling 32 possible offending behaviours (see Appendix A). These categories and descriptions are routinely used in community Forensic Child and Adolescent Mental Health Service (FCAMHS) to gain a better understanding of the YP’s presentation. One point was given for each type of offence committed by each person, as mentioned on available records, or during clinical discussions. Clinical discussions involved a case presentation by the treating clinician in the team, where additional information on the presentation of the referred person was shared.

Offending score did not reflect frequency of offending, but number of different offence types committed. For the purposes of this research, an ‘offence’ was defined as an unlawful act, regardless of whether the person was prosecuted, or convicted. This allowed a wider range of offending behaviour to be captured, after the observation that the referred YP were sometimes not prosecuted due to their young age and vulnerability.

### 2.8. Mental Health and Behavioural Presentation

Data on YP’s mental health and behaviour were similarly extracted from routine data. In this category were included confirmed diagnoses and subclinical presentations based on clinical judgment. This was to overcome challenges in the accurate and timely reporting of medical histories, especially across services. The following conditions were examined and categorised as ‘present/absent’ according to endorsement of symptomatology in clinical records: anxiety, depression, psychosis/psychotic symptoms, obsessive–compulsive disorder, PTSD/complex trauma, emerging personality disorder, conduct disorder (CD), oppositional defiant disorder (ODD), substance use (including alcohol), and other (free-text option). The ‘other’ option included genetic or physical health conditions (e.g., epilepsy), which were not the topic of this research and were therefore not included in analyses.

### 2.9. Plan of Analysis

Prior to any analyses, data were assessed for normality via visual inspection of histograms. Preliminary analyses were conducted to investigate mean differences between the dependent variables—offending scores, ACE scores, mental health, and behavioural presentation, and the two-level independent variable (presence of ASD traits). Independent t-tests were used for normally distributed data while Mann–Whitney U tests were used as the non-parametric equivalent.

Use of substances, conduct disorder (CD), oppositional defiant disorder (ODD), and AHDH are well-established risk factors among forensic youth. Offending score (as a total value and for each offending category separately) was treated as the outcome variable. Due to existing literature citing ethnic minority background as a risk factor for offending, White ethnicity was used as the reference category by which to assess whether being of non-White ethnic background increased offending scores. Therefore, these presentations were chosen as predictors in the regression model.

A Poisson regression was selected as the most appropriate method to investigate the influence of the aforementioned factors on a count outcome variable—the offending scores. A negative binomial regression was chosen as the non-parametric equivalent, for categories that violated the Poisson distribution (sexually harmful behaviour). There was significant under-dispersion observed in the radicalisation, arson and stalking/harassment offending categories, possibly due to the very few YP presenting with such offences. A zero-inflated model was insufficient to provide a good fit for the data, and these categories were therefore excluded from subsequent analyses.

Where the assumption of a Poisson distribution was violated (sexually harmful behaviour), a negative binomial regression was conducted to predict the influence of ASD, ACEs, CD, ODD, ADHD, and substance use on YP’s offending scores, while controlling for age, gender, and ethnic group (White, Black, Asian, mixed, other). A custom estimated value was applied to manage over-dispersion and, although this improved the model, a slight dispersion value of 1.13 was still observed.

All reported *p*-values have been corrected for multiple comparisons using the Benjamin–Hochberg method, which controls for false discovery rate, and is recommended for exploratory research [21].

All statistical analyses were conducted by the primary author (ML), a psychology postgraduate researcher with experience in advanced research methods, and supervised by author RL, who is also an experienced postgraduate psychologist at the doctoral level.

## 3. Results

Data were analysed using IBM SPSS Statistics (version 29.0.1.0). The sample included 272 males (83%), of which 119 (44%) presented with ASD traits. Most of the sample comprised individuals between 15 and 18 years old (64%), while 41% presented with 4 to 6 ACEs. A more detailed description of the YPs’ characteristics is reported on Table 1.

### 3.1. Mean Differences

There was no significant mean difference between the overall offending score of YP with ASD traits (*M* = 5.86, SD = 3.49) and without ASD traits (*M* = 5.92, SD = 3.58), t (324) = −0.17, *p* = 0.867. The only significant comparison was noted for the drug possession/supply offending category, where YP without ASD traits had significantly higher scores, compared with those with ASD traits (U = 11375, *p* = 0.002). Offending score values for YP with and without ASD are presented in Table 2.

YP with ASD traits experienced significantly less ACEs (*M* = 4.81, SD = 2.45) than YP without ASD traits (*M* = 5.60, SD = 2.30), t (323) = −2.99, *p* = 0.004. In terms of their mental health, YP with ASD traits experienced significantly more anxiety (*M* = 0.35, SD = 0.48), than YP without ASD traits (*M* = 0.20, SD = 0.40), t (325) = −2.92, *p* = 0.004. Similarly, those with ASD traits were more likely to present with obsession compulsion disorder traits, U = 13754.5, *p* = 0.025. Significantly more YP without ASD traits used substances (*M* = 0.26, SD = 0.44) when compared with those with ASD traits (*M* = 0.12, SD = 0.33), t (325) = 3.42, *p* = 0.004. All other mental health and behavioural presentations did not differ significantly between the two groups. YPs’ mental health and behavioural presentation is presented in detail in Table 3.

### 3.2. Regression Analyses

The overall model was significant, χ^2^(12) = 46.72, *p* < 0.001. ASD traits did not significantly increase the rate ratio of offending scores (b = 0.104, SE = 0.07, 95% CI [−0.31, 0.24], χ^2^(1) = 2.28, *p* = 0.131). Female gender negatively predicted offending scores, suggesting that male YP had a higher rate ratio of offending scores across offending categories (b = −0.301, SE = 0.09, 95% CI [−0.48, −0.12], χ^2^(1) = 10.89, *p* = 0.004). Minority ethnic background (Black, Asian, mixed, other) significantly increased the rate ratio of offending scores, compared with White ethnic background (b = 0.064, SE = 0.02, 95% CI [0.02, 0.11], χ^2^(1) = 6.68, *p* = 0.018). Further, a greater ACE score significantly increased the rate ratio of offending scores (b = 0.038, SE = 0.01, 95% CI [0.01, 0.07], χ^2^(1) = 7.24, *p* = 0.016).

In terms of mental health and behavioural presentation, substance use (b = 0.323, SE = 0.08, 95% CI [0.17, 0.48], χ^2^(1) = 16.60, *p* = 0.004) and CD (b = 0.176, SE = 0.08, 95% CI [0.02, 0.34], χ^2^(1) = 4.71, *p* = 0.042) were both positive significant predictors of offending across categories. ODD (b = 0.070, SE = 0.12, 95% CI [−0.17, 0.31], χ^2^(1) = 0.32, *p* = 0.572) and ADHD (b = 0.062, SE = 0.07, 95% CI [−0.08, 0.20], χ^2^(1) = 0.77, *p* = 0.442) were both non-significant.

Significant results for the rest of the offending categories are presented in Table 4. Although male gender was found to be the only significant predictor for SHB-related offences, this did not survive significance correction and is therefore not reported.

## 4. Discussion

This exploratory research aimed to identify some of the factors that in combination with ASD traits may increase the risk of more variable offending, in a forensic youth population. To the best of our knowledge, this was the first research to consider the cumulative influence of ACEs, mental health, and neurodiversity on offending behaviour, in a highly diverse and disadvantaged sample of YP. Findings will be discussed with relation to existing literature and their clinical implications.

### 4.1. Autism Spectrum Disorder (ASD) Traits

Almost 43% of YP in this sample presented with ASD traits, with more than 22% having a confirmed ASD diagnosis. This is considerably higher than the general population, from which, at most, up to 17% of participants have been found to have ASD [22]. Considering the presence of ACEs in our sample at higher rates when compared with other studies, it may be possible that some YP are presenting with complex trauma and not necessarily ASD [23]. Many authors have posited the behavioural and structural similarities between PTSD/complex trauma and ASD, including social difficulties and anxiety symptoms that often lead to misdiagnosis [24]. This may reflect a potential lack of specialist training in how trauma may present in children with significant trauma histories, or worse, a degree of hesitancy in attributing certain symptoms to trauma rather than neurodiversity [24]. However, other important parameters to consider are cognitive and attributional biases present in forensic psychiatric practice, which may lead to an overestimation of risk among young people with ASD or other neurodevelopmental disorders [25]. This means that clinicians may be more inclined to refer and ultimately diagnose youth with offending presentations as having ASD. Therefore, a trauma-informed diagnosis can have significant impact in forming the self-identities of YP and prevent the perpetuating legacies of self-blame and worthlessness that are present in unresolved trauma [26].

### 4.2. Substance Use

Substance use was the predictor that significantly increased offending rate ratios for violence, gang involvement, drug possession/supply and theft. Indeed, substance use is higher among offending youth and has been associated with more serious and persistent offending in adolescents [27]. However, research has shown that use of substances is not necessarily related to the initiation of the offending behaviour, suggesting an indirect effect [28]. Some authors have linked earlier and more frequent substance use to the experience of more ACEs, where it may serve as a coping mechanism for those with inadequate support [29]. This appears to hold true for this sample. Previous research on the experience of adversity in this exact sample found an average ACE score of six, which is above the cut-off score of four identified by the Centre for Disease Control and Prevention as an indicator of increased risk of health and other problems [20]. Similarly, the experience of six or more ACEs has been linked to serious, violent, and chronic offenders [30].

### 4.3. Gang Involvement

The combined presence of substance use and higher ACE score was associated with a higher rate ratio of gang involvement in this sample. Longitudinal evidence of juvenile offenders has found ACEs to predict later gang involvement, with this effect largely explained by substance use [31]. Although other authors have linked ACEs to more types of offending behaviour, this was not supported by our findings [32]. Possibly, the qualitative differences in the experience of certain ACEs that cannot be captured by a total score value could better explain the motivations of certain YP to gravitate towards certain types of crimes. Likely, YP who have experienced neglect and abuse may seek acceptance and belonging outside their home environment, increasing the risk of gang participation and offending [33]. Minority ethnic background was also a risk factor for more variable offending in this category. This is in line with existing literature highlighting the over-representation of ethnic minorities in the criminal justice system for ‘gang’-related crimes. This likely points to a wider systemic bias issue where ethnic minorities are more frequently profiled and targeted for gang-related crime [34].

### 4.4. Violence

Regression analyses indicated that comorbid ASD and substance use increased the rate ratio of committing more types of violent offences. The social exclusion experienced by people with ASD (either due to stigma or their social difficulties), in combination with poor affect regulation skills, increase their risk of interpersonal conflict, which they may try to resolve through violence [35]. As services to provide support for youth offenders with ASD are limited, the main responsibility to support YP shifts to clinicians [36]. Interventions to improve cognitive flexibility, the ability to consider alternative perspectives, and to self-regulate, within the context of the therapeutic relationship, may be effective in minimising the risk of resolving conflict through violence among YP with ASD [35].

### 4.5. Clinical Implications

Our findings can help guide the clinical thinking of risk profiles, where there is increased risk of violent offending, particularly among YP presenting with multiple ACEs and ASD. Substance use was highlighted as a key factor in offending in this sample and from various other authors [27]. Several programmes have been trialled to prevent drug use in children and adolescents, including the Drug Abuse Resistance Education programme, though with limited effect [37,38]. While drug education programmes may be useful to some degree, they are possibly insufficient to lead meaningful change on their own and may need to be supplemented [37,39].

Therefore, preventative work is key in reducing youth offending, particularly for community violence and gang-related crime [40]. Although recent evidence highlights the ineffectiveness of various interventions targeted at adult offenders with ASD [41], and considering the aforementioned mixed evidence on substance misuse interventions, multisystemic therapy may be a more promising avenue for reintegrating offending youth in their communities. Multisystemic therapy is a community-based intervention that engages multiple “systems” around the individual, such as their family, school or local community. Evidence on the effectiveness of multisystemic therapy for youth offending in individuals with mental health difficulties or ASD is highly heterogenous [42], but focuses on positive parenting, family cohesion, peer relationships, substance abuse, school attendance, and positive activities during free time to effectively reduce long term recidivism [43].

Considering the majority of our sample experienced significant ACEs, often within parental or family systems that may be unwilling or unable to engage effectively with professionals, multisystemic therapy may be difficult to implement as a stand-alone intervention strategy. It is therefore critical to strengthen integrated service models, particularly between educational settings, mental health, social care services, and the justice system [44]. This will ensure that even YP without a stable home environment are supported by strong alternative systems, reducing the likelihood of future offending.

Furthermore, to enhance the personalisation of multisystemic therapy, we propose the integration of early screening for ACEs, mental health difficulties and substance misuse among YP known to social care and mental health services. This will aid the identification of YP at risk and facilitate timely intervention, before offending behaviours develop. Secondly, multisystemic intervention programmes can include components tailored to address complex trauma or support the needs of those with neurodevelopmental conditions, such as by incorporating trauma-processing and social skills training for YP with ASD.

### 4.6. Future Research

This was an exploratory, cross-sectional study, highlighting the interactions between various demographic variables (e.g., age, gender, ethnicity), and neurodevelopmental and mental health presentation. To further explore the intricate interactions between these variables, future studies should employ longitudinal designs which may shed light into how these factors interact and influence each other. Further, future studies exploring similar risk factors, in communities with better social infrastructure and reduced socioeconomic deprivation could provide further evidence regarding the potential influence of these factors on the offending presentations of youth.

### 4.7. Strengths and Limitations

The present study is the first to investigate the impact of several possible risk factors on youth offending, utilising routine NHS data. It builds on the current literature, reiterating that YP with ASD are not ‘riskier’ but may present with more variable, violent offending, especially if they have certain comorbidities. Further, our research explored risk factors for each offence type, acknowledging the presence of qualitative differences in each type of offence.

Although our sample was sufficient to provide confidence in our statistical findings, the modest effect sizes and wide confidence intervals may suggest the possibility for Type I error, in spite of the statistical corrections applied. As such, findings should be interpreted with caution, warranting replication in larger samples to enhance precision and confidence in our conclusions.

Further, our data only reflect a snapshot of the YP’s offending trajectories and are highly specific to a community-based forensic population with mental health concerns, and significant trauma histories. Although this allowed us to explore a range of potential risk factors, we acknowledge that this might limit the generalisability of our findings to regions with more ethnically homogenous populations or improved social infrastructure. Due to the consultative nature of community Forensic Child and Adolescent Mental Health Service (FCAMHS), and the inability to interview the YP themselves, it is likely that their developmental histories, traumatic experiences, diagnoses, offending behaviours, and other details are subject to bias, or might be under- or over-reported. It is possible that a portion of participants might in fact not fulfil diagnostic criteria for the “suspected” conditions, which could potentially contaminate our sample. However, even YP with “suspected” presentations presented with considerable symptomatology in the assessed reports, providing some confidence that the sample was relatively homogenous in terms of behavioural presentation.

Due to the nature of secondary data available to the authors, it was also not possible to collect frequency data for each offence. Therefore, higher offending scores do not necessarily reflect higher severity or frequency of offending, but a more variable offending presentation. Lastly, some offending categories were excluded from regression analyses due to poor statistical fit.

## 5. Conclusions

Consistent with the current literature, our findings highlight that the presence of comorbid ASD and substance use may increase the rate ratio of violent offending and gang involvement. Thus, our findings highlight how several interpersonal and environmental factors may interact to increase certain YP’s risk for more variable offending, requiring a personalised approach to prevent reoffending. While stand-alone therapy or substance misuse programmes may have limited effectiveness, we emphasise the need for personalised and multisystemic approaches to intervention, that have the potential to contribute to lasting change.

## Figures and Tables

**Table 1:** Participant characteristics.

		ASD * Traits	No ASD * Traits
		Frequency N (%)	Frequency N (%)
Gender	Male	119 (83.8)	153 (82.7)
	Female	23 (16.2)	32 (17.3)
Age (in years)	<11	2 (1.4)	5 (2.7)
	11–14	53 (37.3)	58 (31.4)
	15–18	87 (61.3)	121 (65.4)
	Missing data	0 (0)	1 (0.5)
Learning	Present	52 (36.6)	38 (20.5)
disability	Absent	90 (63.4)	147 (79.5)
ADHD **	Present	65 (45.8)	60 (32.4)
	Absent	77 (54.2)	125 (67.6)
Ethnic	White	67 (47.3)	71 (38.4)
background	Black	40 (28.2)	48 (25.9)
	Asian	10 (7)	21 (11.4)
	Mixed	15 (10.5)	26 (14.1)
	Other	7 (4.9)	18 (9.7)
	Missing data	3 (2.1)	1 (0.5)
Offending score	0–4	60 (42.3)	76 (36)
	5–9	56 (39.5)	79 (42.7)
	10–15	24 (16.8)	29 (19.4)
	16+	1 (0.7)	1 (0.5)
	Missing data	1 (0.7)	0 (0)
Adverse	0–3	44 (31)	33 (17.8)
childhood	4–6	58 (40.3)	77 (41.6)
experiences	7–10	39 (28)	74 (40.1)
	Missing data	1 (0.7)	1 (0.5)

Note. Frequencies of main sample characteristics for YP with or without ASD traits. YP with ASD traits had a confirmed diagnosis of ASD or awaiting/completing a diagnostic assessment. * ASD = autism spectrum disorder; ** ADHD = attention deficit hyperactivity disorder.

**Table 2:** Mean offending scores.

	ASD * Traits		No ASD ** Traits	
Offending Category	Range	Mean (SD)	Range	Mean (SD)
Violence	0–6	2.31 (1.42)	0–5	1.85 (1.39)
Sexually harmful behaviour	0–4	0.82 (1.06)	0–3	0.62 (0.90)
Drug possession/supply	0–1	0.08 (0.28)	0–2	0.24 (0.49)
Gang involvement	0–2	0.24 (0.52)	0–3	0.59 (0.81)
Radicalisation	0–2	0.08 (0.35)	0–3	0.05 (0.31)
Stalking/harassment	0–2	0.07 (0.28)	0–2	0.09 (0.32)
Theft	0–1	0.18 (0.39)	0–1	0.30 (0.46)
Arson	0–1	0.15 (0.36)	0–1	0.07 (0.26)

Note. Table of mean offending scores and standard deviations for each of the assessed offending categories for YP with or without ASD traits. YP with ASD traits had a confirmed diagnosis of ASD or awaiting/completing a diagnostic assessment. For offending presentation, one point was given for each type of offence committed by each participant. Range refers to the minimum and maximum possible score for each offending category, based on the more specific offending presentations detailed in Appendix A. * ASD = autism spectrum disorder; ** ADHD = attention deficit hyperactivity disorder.

**Table 3:** Mental health and behavioural presentation.

		ASD * Traits	No ASD * Traits
Variable	Category	N (%)	N (%)
Anxiety	Present	49 (65.5)	37 (20)
	Absent	93 (34.5)	148 (80)
Depression	Present	34 (23.9)	36 (19.5)
	Absent	108 (76.1)	149 (80.5)
Psychosis/psychotic symptoms	Present	15 (10.6)	20 (10.8)
	Absent	127 (89.4)	165 (89.2)
Obsessive–compulsive disorder	Present	9 (6.3)	3 (1.6)
	Absent	133 (93.7)	182 (98.4)
PTSD **/complex trauma	Present	35 (24.6)	45 (24.3)
	Absent	107 (75.4)	140 (75.7)
Emerging personality disorder	Present	2 (1.4)	9 (4.9)
	Absent	140 (98.6)	176 (95.1)
Conduct disorder	Present	26 (18.3)	34 (18.4)
	Absent	116 (81.7)	151 (81.6)
Oppositional defiant disorder	Present	16 (11.3)	10 (5.4)
	Absent	126 (88.7)	175 (94.6)
Substance use (inc. alcohol)	Present	17 (12)	49 (26.5)
	Absent	125 (88)	136 (73.5)

Note. Number of participants with diagnosed or suspected mental health conditions for YP with or without ASD traits. YP with ASD traits had a confirmed diagnosis of ASD or awaiting/completing a diagnostic assessment. * ASD = autism spectrum disorder; ** PTSD = post-traumatic stress disorder. Values are based on clinical judgment and not necessarily diagnosis.

**Table 4:** Significant regression results based on offending category.

Predictor Variable	*b*	*SE*	95% CI	p *
**Violence**				
Autism spectrum disorder traits	0.25	0.08	[0.08, 0.41]	0.036
*χ* ^2^	36.88			
*Df*	12			
**Gang involvement**				
Autism spectrum disorder traits	−0.53	0.21	[−0.93, −0.12]	0.002
Adverse childhood experiences score	0.12	0.04	[0.04, 0.19]	0.007
Substance use	0.76	0.18	[0.40, 1.12]	0.004
Gender ^a^	−0.86	0.30	[−1.45, −0.28]	0.008
Age	0.20	0.57	[0.09, 0.31]	0.004
Ethnic background ^b^				
Black	0.67	0.22	[0.25, 1.10]	0.006
Asian	0.70	0.31	[0.09, 1.31]	0.036
Mixed	0.90	0.26	[0.39, 1.41]	0.004
*χ* ^2^	82.22			
*Df*	12			
**Drug possession/supply**				
Substance use	1.49	0.29	[0.91, 2.01]	0.012
Age	0.30	0.10	[0.09, 0.49]	0.014
Ethnic background ^b^				
Black	1.13	0.39	[0.38, 1.89]	0.014
Mixed	1.30	0.45	[0.41, 2.19]	0.014
Other	1.22	0.44	[0.36, 2.08]	0.014
*χ* ^2^	70.53			
*Df*	12			
**Theft**				
Substance use	0.89	0.25	[0.40, 1.39]	0.012
*χ* ^2^	29.76			
*Df*	12			

Note. Results of statistically significant regression analyses for each of the offending categories (shown in bold). In bold are presented the offending categories analysed, with significant predictor variables presented underneath. SE = standard error; CI = confidence interval; df = degrees of freedom. ^a^ Female gender was used as the reference category. ^b^ White ethnic background was used as the reference category. * *p* = *p*-values are corrected based on the Benjamin–Hochberg method, significance set at α = 0.05.

## Data Availability

No new data were created or analysed in this study. Data sharing is not applicable to this article.

## Abbreviations

The following abbreviations are used in this manuscript:

ACE	Adverse childhood experience
ADHD	Attention deficit hyperactivity disorder
ASD	Autism spectrum disorder
CD	Conduct disorder
FCAMHS	Forensic Childhood and Adolescent Mental Health Service
PTSD	Post-traumatic stress disorder
SHB	Sexually harmful behaviour
YP	Young people

## Appendix A

Information collected for outcome monitoring purposes in the Forensic Child and Adolescent Mental Health Service (FCAMHS), outlining the neurodevelopmental profile, mental health and behavioural presentation, offending presentation, and adverse childhood experiences, for each referred person.

Neurodevelopmental presentation, as follows:

- Confirmed or suspected autism spectrum disorder diagnosis.
- Confirmed or suspected learning disability diagnosis.
- Diagnosed learning difficulty.
- Confirmed or suspected attention deficit hyperactivity disorder diagnosis.

Mental health and behavioural presentation, based on diagnosis or clinical judgment.

- Anxiety.
- Depression.
- Psychosis/psychotic symptoms.
- Obsessive–compulsive disorder.
- Post-traumatic stress disorder/complex trauma.
- Personality disorder/emerging personality disorder.
- Conduct disorder.
- Oppositional defiant disorder.
- Substance use.
- Other (specify).

Offending presentation (regardless of prosecution).

Violence.

- Physical violence towards humans.
- Use of a weapon.
- Possession of a weapon.
- Threats of violence.
- Thoughts of violence.
- Damage to property.
- Viewing violent material.
- Murder/attempted murder.
- Animal cruelty/abuse/killed animals.

Drug possession/supply.

- Possession of drugs.
- Possession of drugs with intent to supply.

Sexually harmful behaviour.

- Possessing/viewing content of minors.
- Possessing/viewing violent pornography.
- Sexual assault, including rape.
- Attempted sexual assault.
- Verbal threats of sexual assault.
- Inappropriate sexual behaviour (interest in nappies, animals, etc).

Gang involvement.

- Violence related to gang involvement.
- Child criminal exploitation linked to gang activity.
- Risk of child criminal exploitation linked to gang activity.
- Risk of child criminal exploitation related to drug possession/supply.

Radicalisation.

- Terrorism.
- Extremism.
- Risk of radicalisation.
- Theft.
- Robbery/attempted robbery.

Fire setting/arson.

Stalking/harassment.

Adverse childhood experiences.

- Verbal abuse.
- Physical abuse.
- Sexual abuse.
- Physical neglect.
- Emotional neglect.
- Domestic violence.
- Parental separation.
- Household mental health.
- Household substance misuse.
- Household incarceration.

## References

[1] Chester V., Bunning K., Tromans S., Alexander R., Langdon P. (2022). The Prevalence of Autism in the Criminal Justice System: A Systematic Review. BJPsych Open.

[2] Helverschou S.B., Steindal K., Nøttestad J.A., Howlin P. (2018). Personal experiences of the Criminal Justice System by individuals with autism spectrum disorders. Autism.

[3] Im D.S. (2016). Template to Perpetrate: An Update on Violence in Autism Spectrum Disorder. Harv. Rev. Psychiatry.

[4] Cheely C.A., Carpenter L.A., Letourneau E.J., Nicholas J.S., Charles J., King L.B. (2012). The prevalence of youth with autism spectrum disorders in the criminal justice system. J. Autism Dev. Disord..

[5] van Buitenen N., Meijers J., van den Berg C.J.W., Harte J.M. (2021). Risk factors of violent offending in mentally ill prisoners with autism spectrum disorders. J. Psychiatr. Res..

[6] Latvala A., Tideman M., Søndenaa E., Larsson H., Butwicka A., Fazel S., Lichtenstein P. (2023). Association of intellectual disability with violent and sexual crime and victimization: A population-based cohort study. Psychol. Med..

[7] McDonnell C.G., Boan A.D., Bradley C.C., Seay K.D., Charles J.M., Carpenter L.A. (2019). Child maltreatment in autism spectrum disorder and intellectual disability: Results from a population-based sample. J. Child Psychol. Psychiatry.

[8] Gunasekaran S., Chaplin E. (2012). Autism spectrum disorders and offending. Adv. Ment. Health Intellect. Disabil..

[9] Lerner M.D., Haque O.S., Northrup E.C., Lawer L., Bursztajn H.J. (2012). Emerging perspectives on adolescents and young adults with high-functioning autism spectrum disorders, violence, and criminal law. J. Am. Acad. Psychiatry Law.

[10] Heeramun R., Magnusson C., Gumpert C.H., Granath S., Lundberg M., Dalman C., Rai D. (2017). Autism and Convictions for Violent Crimes: Population-Based Cohort Study in Sweden. J. Am. Acad. Child Adolesc. Psychiatry.

[11] Payne K.-L., Maras K.L., Russell A.J., Brosnan M.J. (2021). Are Mental Health, Family and Childhood Adversity, Substance Use and Conduct Problems Risk Factors for Offending in Autism?. J. Autism Dev. Disord..

[12] Felitti V.J., Anda R.F., Nordenberg D., Williamson D.F., Spitz A.M., Edwards V., Koss M.P., Marks J.S. (1998). Relationship of childhood abuse and household dysfunction to many of the leading causes of death in adults. The Adverse Childhood Experiences (ACE) Study. Am. J. Prev. Med..

[13] Bevilacqua L., Kelly Y., Heilmann A., Priest N., Lacey R.E. (2021). Adverse childhood experiences and trajectories of internalizing, externalizing, and prosocial behaviors from childhood to adolescence. Child Abus. Negl..

[14] Jahanshahi B., Murray K., McVie S. (2022). ACEs, Places and Inequality: Understanding the Effects of Adverse Childhood Experiences and Poverty on Offending in Childhood. Br. J. Criminol..

[15] Census 2021 Reports. https://apps.london.gov.uk/census-2021-reports/#/country-of-birth.

[16] Household and Resident Characteristics, England and Wales—Office for National Statistics. https://www.ons.gov.uk/peoplepopulationandcommunity/householdcharacteristics/homeinternetandsocialmediausage/bulletins/householdandresidentcharacteristicsenglandandwales/census2021#household-deprivation.

[17] Lane R., D’Souza S., Singleton R., Hindley N., Bevington D., White O., Jacob J., Wheeler J., Edbrooke-Childs J. (2023). Characteristics of young people accessing recently implemented Community Forensic Child and Adolescent Mental Health Services (F:CAMHS) in England: Insights from national service activity data. Eur. Child Adolesc. Psychiatry.

[18] ‘Ethnicity’. NHS Digital. https://digital.nhs.uk/data-and-information/data-collections-and-data-sets/data-sets/mental-health-services-data-set/submit-data/data-quality-of-protected-characteristics-and-other-vulnerable-groups/ethnicity.

[19] Autism Assessment Waiting Times. https://www.autism.org.uk/what-we-do/news/autism-assessment-waiting-times-2.

[20] Marshall S., Loizidou M., Busse A., McRae R. (2024). Adverse childhood experiences in a London-based forensic CAMHS population. Forensic Update.

[21] Waite T.A., Campbell L.G. (2006). Controlling the false discovery rate and increasing statistical power in ecological studies. Ecoscience.

[22] CDC ‘Data and Statistics on Autism Spectrum Disorder’, Autism Spectrum Disorder (ASD). https://www.cdc.gov/autism/data-research/index.html.

[23] Kildahl A.N., Storvik K., Wächter E.C., Jensen T., Ro A., Haugen I.B. (2024). Distinguishing between autism and the consequences of early traumatisation during diagnostic assessment: A clinical case study. Adv. Autism.

[24] Rowland D. (2020). Differential diagnosis of autism: A causal analysis. J. Neurol. Neurophysiol..

[25] Buongiorno L., Mele F., Petroni G., Margari A., Carabellese F., Catanesi R., Mandarelli G. (2025). Cognitive biases in forensic psychiatry: A scoping review. Int. J. Law Psychiatry.

[26] McCormack L., Thomson S. (2017). Complex trauma in childhood, a psychiatric diagnosis in adulthood: Making meaning of a double-edged phenomenon. Psychol. Trauma Theory Res. Pract. Policy.

[27] Mulvey E.P., Schubert C.A., Chassin L. (2010). Substance Use and Delinquent Behavior Among Serious Adolescent Offenders. Juvenile Justice Bulletin.

[28] Lennings C.J., Copeland J., Howard J. (2003). Substance use patterns of young offenders and violent crime. Aggress. Behav..

[29] Oei A., Chu C.M., Li D., Ng N., Yeo C., Ruby K. (2021). Relationship between Adverse Childhood Experiences and substance use in youth offenders in Singapore. Child Abus. Negl..

[30] Fox B.H., Perez N., Cass E., Baglivio M.T., Epps N. (2015). Trauma changes everything: Examining the relationship between adverse childhood experiences and serious, violent and chronic juvenile offenders. Child Abus. Negl..

[31] Wolff K.T., Baglivio M.T., Klein H.J., Piquero A.R., DeLisi M., Howell J.C. (2020). (Buddy) Adverse Childhood Experiences (ACEs) and Gang Involvement Among Juvenile Offenders: Assessing the Mediation Effects of Substance Use and Temperament Deficits. Youth Violence Juv. Justice.

[32] Baglivio M.T., Wolff K.T., Piquero A.R., Epps N. (2015). The Relationship between Adverse Childhood Experiences (ACE) and Juvenile Offending Trajectories in a Juvenile Offender Sample. J. Crim. Justice.

[33] Estrada J.N., Huerta A.H., Hernandez E., Hernandez R.A., Kim S.W., Shapiro H. (2018). Socio-Ecological Risk and Protective Factors for Youth Gang Involvement. The Wiley Handbook on Violence in Education.

[34] Byrne B., Alexander C., Khan O., Nazroo J., Shankley W. (2020). Ethnicity, Race and Inequality in the UK: State of the Nation.

[35] Murphy D. (2010). Understanding offenders with autism-spectrum disorders: What can forensic services do? Commentary on… Asperger Syndrome and Criminal Behaviour. Adv. Psychiatr. Treat..

[36] Howlin P., Goode S., Hutton J., Rutter M. (2004). Adult outcome for children with autism. Child Psychol. Psychiatry.

[37] Valente J.Y., Galvão P.P.d.O., Gusmoes J.D.S.P., Sanchez Z.M. (2022). Revisão sistemática sobre o efeito do programa escolar de prevenção ao uso de drogas Keepin’ it REAL: Traduzido e implementado no Brasil pelo PROERD. Ciênc. Saúde Coletiva.

[38] Brown J.H. (2001). Youth, drugs and resilience education. J. Drug Educ..

[39] Valente J.Y., Sanchez Z.M. (2022). Short-Term Secondary Effects of a School-Based Drug Prevention Program: Cluster-Randomized Controlled Trial of the Brazilian Version of DARE’s Keepin’ it REAL. Prev. Sci..

[40] Garbarino J., Governale A., Nesi D. (2020). Vulnerable children: Protection and social reintegration of child soldiers and youth members of gangs. Child Abus. Negl..

[41] Salter J., Blainey S. (2024). The effectiveness of interventions for offending behaviours in adults with autism spectrum disorders (ASD): A systematic PRISMA review. BMC Psychol..

[42] Littell J.H., Pigott T.D., Nilsen K.H., Green S.J., Montgomery O.L.K. (2021). Multisystemic Therapy^®^ for social, emotional, and behavioural problems in youth age 10 to 17: An updated systematic review and meta-analysis. Campbell Syst. Rev..

[43] Bjørknes R., Hukkelberg S., Taraldsen K., Høstmælingen A.T. (2024). Does Multisystemic Therapy Change Criminogenic Risk Factors? A 10-Year Study Among Norwegian Youths With and Without Offenses. Crime Delinq..

[44] Klymkiw D.F., Day D.M., Henderson J.L., Hawke L.D. (2024). What do justice-involved youth want from integrated youth services? A conjoint analysis. J. Can. Acad. Child Adolesc. Psychiatry.

